# Psychological perceptions to behavioral engagement: testing a mediated structural model of smart interactive product use and subjective wellbeing in older adults

**DOI:** 10.3389/fpsyg.2026.1861208

**Published:** 2026-10-28

**Authors:** Tianyu Wu, Tianlu Zhu, Yiqian Zhao, Cengjuan Wu

**Affiliations:** 1 School of Art, Anhui University, Hefei, China; 2 Department of Mechanical Engineering, North China Electric Power University, Baoding, China; 3 School of Design, ShangHai Jiao Tong University, Shanghai, China; 4 School of Design Art and Media, Nanjing University of Science and Technology, Nanjing, China

**Keywords:** behavioral engagement, older adults, psychological need satisfaction, psychological perceptions, smart interactive products, structural equation modeling, subjective wellbeing

## Abstract

**Background:**

As population aging and digitalization advance, smart interactive products are becoming part of older adults’ everyday lives. Although these technologies are expected to support independence, communication, and wellbeing, existing findings remain mixed. Prior research has focused mainly on technology acceptance or general use, with limited attention to how older adults’ psychological perceptions are translated into behavioral engagement and, in turn, subjective wellbeing. This study therefore proposes the Technology Engagement and Wellbeing (TEW) model to explain this mechanism.

**Methods:**

A questionnaire survey was conducted among adults aged 60 years and above with prior experience using smart interactive products. Of 2,686 collected questionnaires, 2,635 valid responses were retained. Psychological perceptions were represented by perceived usefulness, technology self-efficacy, and technology anxiety. Behavioral engagement was modeled as a multidimensional construct including usage frequency, functional diversity, and interactive depth. Structural equation modeling and bootstrap analysis were used to test the hypothesized relationships, and digital literacy was examined as a moderator.

**Results:**

The structural model showed good fit to the data (*χ*^2^/df = 2.618, CFI = 0.979, TLI = 0.977, RMSEA = 0.025). Psychological perceptions were positively associated with behavioral engagement (*β* = 0.651, *p* < 0.001) and psychological need satisfaction (*β* = 0.441, *p* < 0.001). Behavioral engagement was positively associated with psychological need satisfaction (*β* = 0.255, *p* < 0.001) and subjective wellbeing (*β* = 0.115, *p* < 0.001), while psychological need satisfaction was positively associated with subjective wellbeing (*β* = 0.422, *p* < 0.001). The serial indirect association of psychological perceptions with subjective wellbeing through behavioral engagement and psychological need satisfaction was significant [*β* = 0.070, 95% CI (0.058, 0.084)]. Digital literacy also significantly moderated the association between psychological perceptions and behavioral engagement (*β* = 0.390, *p* < 0.001).

**Conclusion:**

The wellbeing value of smart interactive products lies not in access alone, but in whether positive psychological perceptions can be translated into active engagement that supports autonomy, competence, and relatedness. The TEW model provides an integrated framework for understanding how smart interactive product use becomes beneficial in later life.

## 1 Introduction

Population aging and digitalization are advancing simultaneously, making smart interactive products increasingly relevant to older adults’ daily lives (Kebede et al., 2022). Technologies such as smartphones, voice assistants, wearable devices, and smart health systems are expected to support independence, communication, and everyday wellbeing in later life (Marziali et al., 2024). Understanding whether and how such technologies contribute to older adults’ subjective wellbeing has therefore become an important research issue.

Existing studies suggest that digital technology use may improve social connectedness, access to information, and support for daily and health-related activities among older adults (Liu and Li, 2024). However, the evidence remains mixed. While some studies report positive associations between technology use and wellbeing, others show that older adults often experience anxiety, low confidence, or difficulty when interacting with digital technologies (An et al., 2024). These barriers may limit meaningful engagement and reduce the potential benefits of technology use.

One reason for these inconsistent findings is that prior research has often treated technology use too broadly (Liu and Li, 2024). Much of the literature has focused on technology acceptance, adoption intention, or general use status, but has paid less attention to how psychological perceptions are translated into actual behavioral engagement with smart interactive products (Bertolazzi et al., 2024). This distinction matters because occasional use is not equivalent to frequent, diverse, and in-depth engagement (Du et al., 2026).

Another limitation is that psychological antecedents of technology use and wellbeing outcomes have often been studied separately. Research has shown that perceived usefulness, technology self-efficacy, and technology anxiety are important in shaping older adults’ responses to digital technologies (Money et al., 2024). At the same time, studies on wellbeing have often examined whether technology use is associated with positive outcomes without clearly explaining the mechanism through which these outcomes emerge (Lu, 2024). As a result, the literature still lacks an integrated explanation of how older adults’ psychological perceptions of smart interactive products are translated into behavioral engagement and how such engagement subsequently contributes to subjective wellbeing.

To address this gap, the present study proposes the Technology Engagement and Wellbeing (TEW) model by integrating the Technology Acceptance Model, Self-Efficacy Theory, and Self-Determination Theory. The model conceptualizes psychological perceptions through perceived usefulness, technology self-efficacy, and technology anxiety, and proposes that these perceptions influence subjective wellbeing indirectly through behavioral engagement and psychological need satisfaction. Behavioral engagement is expected to promote the satisfaction of autonomy, competence, and relatedness, which in turn enhances subjective wellbeing. In addition, digital literacy is incorporated as a moderator of the relationship between psychological perceptions and behavioral engagement.

Accordingly, this study examines how older adults’ psychological perceptions of smart interactive products influence behavioral engagement, how behavioral engagement contributes to psychological need satisfaction, and how these processes ultimately relate to subjective wellbeing. It also tests whether digital literacy strengthens the relationship between psychological perceptions and behavioral engagement. By doing so, this study provides an integrated framework for understanding how smart interactive product use may become meaningful and beneficial in later life.

Furthermore, the present study focuses specifically on older adults with prior experience using smart interactive products. The TEW model examined here is intended to explain how technology-related psychological perceptions are translated into behavioral engagement and subsequent psychological benefits after initial exposure to or use of smart interactive products. It is not intended to explain the transition from complete technology non-use to initial adoption.

The remainder of this article is organized as follows. Section 2 reviews the relevant literature, develops the theoretical basis of the TEW model, and presents the research hypotheses. Section 3 describes the methodology, including participants, measures, and data analysis procedures. Section 4 reports the empirical results. Section 5 discusses the findings, implications, and limitations. Section 6 concludes the article.

## 2 Theoretical framework

### 2.1 Technology use and wellbeing in older adults

Population aging and digitalization are reshaping the everyday lives of older adults at the same time. Recent research consistently shows that digital technologies can support social connectedness, aging in place, access to information, and aspects of wellbeing in later life, but older adults remain disproportionately excluded from these benefits (Liu et al., 2023). A recent scoping review of 96 studies showed that research on older adults’ digital engagement has grown rapidly, yet the evidence is still concentrated on initial adoption rather than on sustained and meaningful engagement (Kebede et al., 2022). The same review also found that environmental constraints, beliefs about capabilities, and physical and cognitive capability changes are the most frequently reported determinants of older adults’ digital engagement, indicating that technology use in later life is shaped by far more than access alone (Huang et al., 2023).

Empirical studies have provided several reasons to expect a positive association between digital technology use and subjective wellbeing in later life. Kebede et al. (2022) noted that older adults often perceive digital technologies as beneficial when these technologies help restore independence, support decision-making, and strengthen social connection. Empirical studies also suggest that technology use may be linked to life satisfaction through concrete social mechanisms rather than through a vague “technology effect.” For example, Cheng et al. (2024) found that internet use was positively associated with life satisfaction among Chinese older adults, with offline social interaction serving as a significant mediator. Similarly, Ractham et al. (2022) showed that satisfaction derived from older adults’ social media activities was positively associated with life satisfaction across multiple life domains, including family, community, health, leisure, and self-development. These studies are useful because they move beyond the simple claim that technology is beneficial and instead show that benefits emerge when technology becomes embedded in social and everyday practices.

At the same time, recent evidence does not support a uniformly positive conclusion. Reviews focusing on loneliness and social isolation show that the effects of technology-based interventions are mixed and often weaker than popular discourse suggests. Sen et al. (2022) in a systematic review, argued that technology can support social wellbeing in older adults, especially when interventions are tailored and accompanied by community-level support and training. However, a meta-analysis of randomized controlled trials by Jin et al. (2021) found that technology-based interventions had little or no overall effect on reducing loneliness in older adults, and the authors explicitly cautioned against treating technology itself as the solution. Their conclusion was sharper: technology may function as a tool for maintaining connection, but its effects depend on users’ needs, abilities, and contextual resources (Jin et al., 2021). A recent review on voice assistants reached a similar conclusion. Although voice assistants may appear promising because they reduce some interaction barriers through more natural interfaces, most existing studies still focus on usability, acceptability, or user experience, while direct evidence on loneliness reduction or broader wellbeing outcomes remains limited (Marziali et al., 2024).

A key reason for these mixed findings lies in how technology use has been conceptualized. Much of the literature still treats technology use as a broad behavioral outcome, such as adoption, access, or general internet use (Pejić Bach et al., 2023). This approach is analytically limited because it collapses qualitatively different forms of use into a single indicator. An older adult who occasionally uses one digital service for a routine task is not behaviorally equivalent to an older adult who frequently uses multiple functions, explores new features, and integrates digital tools into health management or social communication. Kebede et al. (2022) explicitly argued that sustained digital engagement remains underexamined relative to initial adoption. Recent mechanism-oriented work has begun to move beyond this simplification. For example, a 2026 JMIR study modeled digital engagement as a structured construct and showed that its effects operate through mediating mechanisms such as digital health literacy and social support (Hansen et al., 2025). This line of work indicates that deeper forms of engagement are conceptually and empirically distinguishable from simple use status.

Another unresolved issue is that psychological antecedents and wellbeing outcomes are still too often studied separately. On the one hand, recent studies clearly show that psychological variables matter for older adults’ digital behavior. An et al. (2024) demonstrated that technology anxiety, self-efficacy, and perceived usefulness are central to older adults’ intention to use digital public services, with perceived usefulness emerging as especially important in their model. Money et al. (2024) found that adults aged over 75 years encountered barriers and facilitators to engagement with web-based services that were closely tied to confidence, support, and perceived ability. Knotnerus et al. (2024) similarly reported that older adults’ experiences with a digital health platform were strongly conditioned by digital literacy, usability, and contextual support. On the other hand, these studies mostly stop at intention, access, or service experience, whereas wellbeing studies often measure technology use without adequately specifying the psychological process through which engagement becomes beneficial (Kim et al., 2023). As a result, the literature still does not explain clearly how older adults’ psychological perceptions of smart interactive products are translated into actual behavioral engagement and how that engagement subsequently contributes to subjective wellbeing. To clarify the current state of the literature, Table 1 summarizes representative recent studies on digital technology use and wellbeing among older adults.

**Table 1:** Representative recent studies on digital technology use and wellbeing among older adults.

Reference	Study type	Technology context	Core variables	Main contribution	Limitation relevant to this study
Kebede et al. (2022)	Scoping review	General digital engagement	Nonuse, adoption, sustained engagement; barriers and facilitators	Shows that older adults’ digital engagement is a staged process shaped by capability beliefs, context, and support	Focuses on mapping determinants of engagement rather than linking them to subjective wellbeing
Money et al. (2024)	Qualitative study	Web-based public services	Barriers, facilitators, confidence, support, digital behavior among adults aged >75 years	Highlights that confidence, support, and perceived ability remain central barriers in very old adults	Does not test a structural mechanism linking perceptions, engagement, and wellbeing
Knotnerus et al. (2024)	Qualitative study	Digital health platform in general practice	User experience, digital literacy, barriers and facilitators	Shows that digital health use depends strongly on literacy, usability, and contextual support	Focuses on service experience rather than broader wellbeing outcomes
An et al. (2024)	Empirical SEM study	Digital public services	Technology anxiety, self-efficacy, perceived usefulness, behavioral intention	Demonstrates that psychological factors strongly shape older adults’ technology-related intention	Outcome is intention rather than multidimensional behavioral engagement
Cheng et al. (2024)	Empirical study	Internet use	Internet use, life satisfaction, online and offline social interaction	Shows that internet use is positively associated with life satisfaction and that offline social interaction mediates this link	Treats technology use relatively broadly and does not distinguish engagement depth
Furlan and Meggiolaro (2025)	Empirical study	Internet activities in later life	Types of online activities, living arrangements, life satisfaction	Shows that different online activities relate differently to older adults’ life satisfaction	Does not model psychological antecedents of technology engagement
Hansen et al. (2025)	Systematic review and meta-analysis	Digital interventions for loneliness and social isolation	Intervention effects in older adults	Shows that digital interventions do not produce uniformly positive effects	Confirms mixed outcomes but does not explain why some older adults benefit more than others
Du et al. (2026)	Empirical moderated mediation study	Digital engagement	Digital engagement, digital health literacy, social support, cognitive function	Shows that digital engagement can be modeled as a structured mechanism operating through mediators	Outcome is cognitive function rather than subjective wellbeing, and antecedent perceptions are underdeveloped

As shown in Table 1, recent studies have generated important evidence on digital technology use in later life, but three limitations remain. First, many studies still operationalize technology use in a broad way, making it difficult to distinguish superficial use from deeper engagement. Second, although several studies identify psychological variables such as self-efficacy, anxiety, and perceived usefulness, these factors are often linked to behavioral intention or service adoption rather than to multidimensional engagement. Third, studies examining wellbeing outcomes usually report social or functional benefits without specifying the psychological and behavioral mechanism through which those benefits emerge. These limitations indicate the need for a more integrated framework that links psychological perceptions, behavioral engagement, and subjective wellbeing in a single explanatory model.

### 2.2 The technology engagement and wellbeing (TEW) model

To address the limited understanding of how older adults’ technology-related perceptions are translated into meaningful engagement and subsequent wellbeing, the present study proposes the TEW (Technology Engagement and Wellbeing) model, which integrates three analytical layers: cognitive appraisal, behavioral engagement, and psychological benefit. The model draws on the Technology Acceptance Model, Self-Efficacy Theory, and Self-Determination Theory to explain how older adults form perceptions of smart interactive products, how these perceptions are converted into sustained engagement, and why such engagement may enhance wellbeing. Figure 1 presents the conceptual structure of the TEW model. Specifically, the model proposes that older adults’ psychological perceptions of smart interactive products influence subjective wellbeing indirectly through behavioral engagement and psychological need satisfaction, with digital literacy moderating the link between psychological perceptions and behavioral engagement.

**Figure 1:**
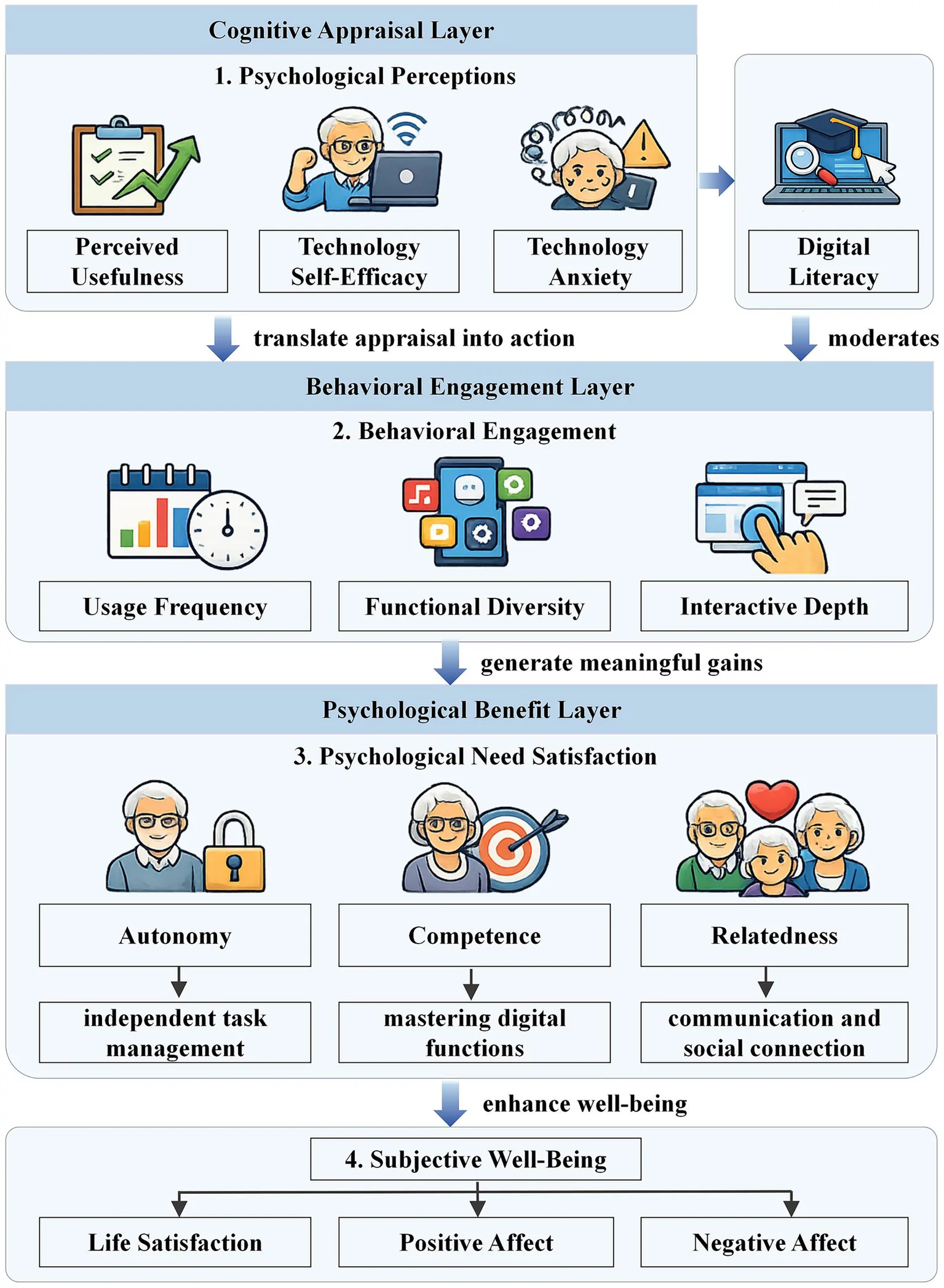
Conceptual framework of the TEW model.

At the cognitive appraisal layer, TAM provides the theoretical basis for perceived usefulness. Davis (1989)conceptualized perceived usefulness as an individual’s belief that using a technology can improve performance or provide valued functional benefits. Within the present context, perceived usefulness reflects the extent to which older adults consider smart interactive products beneficial for daily activities, communication, safety, or health management. This construct remains relevant in contemporary research on older adults’ technology use. Recent studies have shown that perceived usefulness is closely associated with older adults’ acceptance of digital health services and digital public services (Jokisch et al., 2022; An et al., 2024), while research on smart healthcare devices has similarly identified perceived usefulness as an important technology-related appraisal associated with willingness to use such products (Sun et al., 2024). These findings support the relevance of usefulness-based appraisal beyond conventional information systems and in contemporary smart-product contexts.

At the behavioral engagement layer, Self-Efficacy Theory provides a basis for understanding capability-related perceptions. Self-efficacy refers to individuals’ beliefs regarding their capability to organize and perform the actions required to achieve desired outcomes (Bandura, 1977). Applied to smart interactive products, technology self-efficacy therefore reflects older adults’ confidence in learning, operating, and managing digital functions. Technology anxiety represents a related negative psychological appraisal characterized by discomfort, concern, or anticipated difficulty during technology use. Although technology anxiety is not itself a core construct of Self-Efficacy Theory, recent studies of older adults have consistently shown that self-efficacy and technology anxiety are closely associated with digital technology acceptance and engagement. For example, self-efficacy has been linked to acceptance of digital health services (Jokisch et al., 2022), while technology anxiety and self-efficacy have jointly been identified as important correlates of older adults’ intention to use digital public services (An et al., 2024). Recent evidence further indicates that digital anxiety and perceived capability are closely related to the development of digital literacy among older adults (Niu et al., 2026). The TEW model therefore incorporates technology self-efficacy and technology anxiety alongside perceived usefulness to represent complementary dimensions of older adults’ psychological perceptions.

At the psychological benefit layer, SDT explains the role of psychological need satisfaction. SDT identifies autonomy, competence, and relatedness as fundamental psychological needs associated with optimal functioning and wellbeing (Deci and Ryan, 1985). Autonomy concerns the experience of volition and personal control, competence concerns the experience of effectiveness and capability, and relatedness concerns the experience of meaningful connection with others. These needs are particularly relevant to smart interactive product use in later life. Recent research applying SDT to eHealth has shown that digital technologies may be associated with older adults’ experiences of autonomy, competence, and relatedness when they support independent action, successful technology use, and social connection (Cotter et al., 2024). Related evidence from age-appropriate smart wearable technologies also indicates that technology-related affordances are associated with older adults’ wellbeing through psychologically meaningful experiences (Zhang et al., 2025). Accordingly, the TEW model positions psychological need satisfaction as the proximal psychological link between behavioral engagement and subjective wellbeing.

Integrating these theoretical perspectives, the TEW model specifies a sequential structure in which older adults’ psychological perceptions of smart interactive products are associated with their level of behavioral engagement, reflected in usage frequency, functional diversity, and interactive depth. Greater behavioral engagement is in turn associated with higher psychological need satisfaction, which is subsequently associated with subjective wellbeing. Digital literacy is incorporated as a moderator of the association between psychological perceptions and behavioral engagement because the translation of favorable technology perceptions into actual engagement may differ according to older adults’ digital capabilities. Through this integration, the TEW model connects technology appraisal, capability-related perceptions, behavioral engagement, and psychological need fulfillment within a unified framework for examining smart interactive product use in later life.

### 2.3 Hypotheses development

Based on the TEW model, this study develops hypotheses concerning the relationships among psychological perceptions, behavioral engagement, psychological need satisfaction, subjective wellbeing, and digital literacy.

Older adults’ behavioral engagement with smart interactive products is shaped by how they perceive these technologies (Jokisch et al., 2022). In the present study, psychological perceptions include perceived usefulness, technology self-efficacy, and technology anxiety (Sun et al., 2024). Perceived usefulness provides a reason for use, technology self-efficacy supports confidence in performing digital tasks, and technology anxiety may inhibit interaction by increasing discomfort and anticipated difficulty (Cao et al., 2024). Taken together, these perceptions are expected to be associated with the extent to which older adults actively engage with smart interactive products. Therefore, the following hypothesis is proposed:

*H1*: More favorable psychological perceptions toward smart interactive products are positively associated with greater behavioral engagement among older adults.

Behavioral engagement is further expected to contribute to psychological need satisfaction (Cotter et al., 2024). According to Self-Determination Theory, autonomy, competence, and relatedness are basic psychological needs closely associated with positive functioning. In the present context, more active engagement with smart interactive products may support autonomy by facilitating independent daily activities, competence by enabling successful use and skill development, and relatedness by enhancing communication and connection with others (Zhang et al., 2025). Therefore, the following hypothesis is proposed:

*H2*: Greater behavioral engagement with smart interactive products is positively associated with higher psychological need satisfaction among older adults.

Psychological need satisfaction is expected to be positively associated with subjective wellbeing (Zhang et al., 2025). Self-Determination Theory suggests that individuals experience higher wellbeing when their needs for autonomy, competence, and relatedness are fulfilled (Cotter et al., 2024). For older adults, these needs are particularly relevant because they are closely tied to independence, self-confidence, and social connection in later life. Therefore, the following hypothesis is proposed:

*H3*: Higher psychological need satisfaction is positively associated with greater subjective wellbeing among older adults.

The preceding relationships also suggest an indirect mechanism linking psychological perceptions and subjective wellbeing. Positive psychological perceptions alone are unlikely to enhance wellbeing unless they are translated into actual engagement with smart interactive products. More active engagement may then support the satisfaction of autonomy, competence, and relatedness, and these fulfilled needs are expected to contribute to higher subjective wellbeing (Shi et al., 2023). In this sense, behavioral engagement and psychological need satisfaction jointly explain how older adults’ psychological perceptions toward smart interactive products are converted into wellbeing outcomes. Therefore, the following hypothesis is proposed:

*H4*: There is a significant serial indirect association between psychological perceptions and subjective wellbeing through behavioral engagement and psychological need satisfaction.

In addition to this indirect mechanism, the strength of the relationship between psychological perceptions and behavioral engagement may depend on older adults’ digital literacy (Quinn et al., 2024). Older adults with higher digital literacy are more likely to understand digital interfaces, handle routine operations, and convert favorable perceptions into actual use. By contrast, even when psychological perceptions are positive, lower digital literacy may weaken the translation of those perceptions into sustained engagement. Therefore, the following hypothesis is proposed:

*H5*: Digital literacy moderates the association between psychological perceptions and behavioral engagement, such that the positive association is stronger among older adults with higher digital literacy.

The five hypothesized relationships proposed above are summarized in Figure 2.

**Figure 2:**
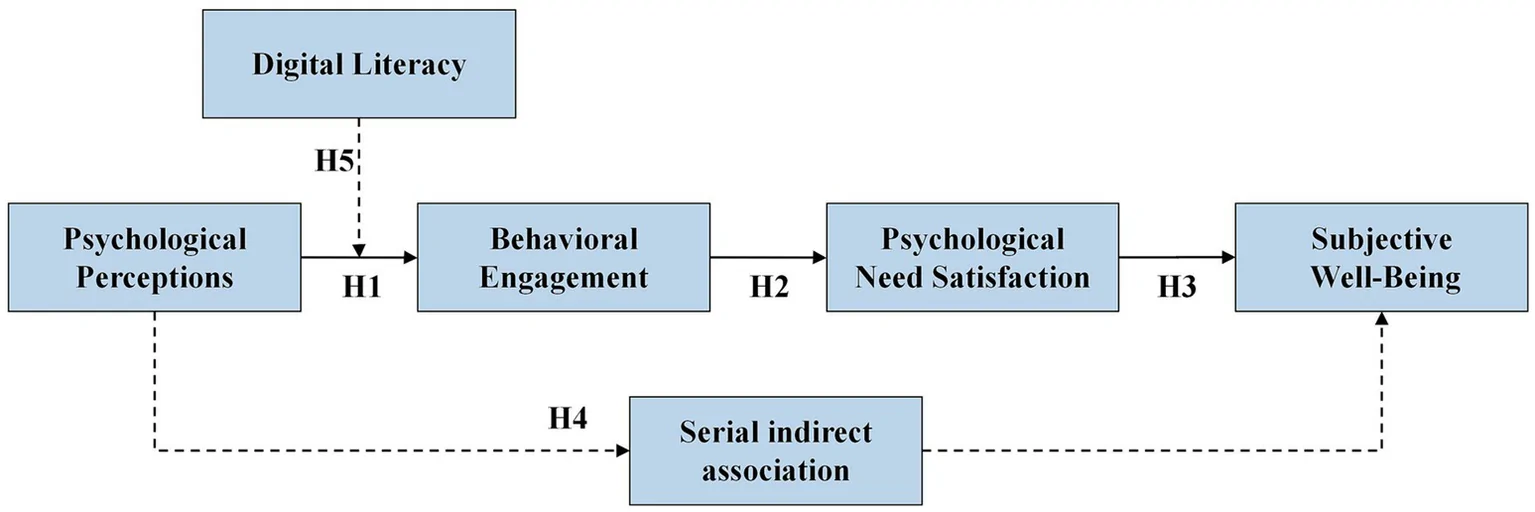
Proposed hypotheses and structural relationships in the TEW model.

## 3 Method

### 3.1 Participants and procedure

Data for this study were collected through a questionnaire survey targeting older adults with experience using smart interactive products. To ensure that respondents were relevant to the research context, participants were required to meet the following criteria: they had to be aged 60 years or above and have prior experience using at least one type of smart interactive product, such as smartphones, wearable devices, voice assistants, or smart home systems (Kim and Choudhury, 2021). Data collection was conducted over approximately 1 month, from mid March to mid April 2026. Online questionnaires were administered through Questionnaire Star and distributed through social platforms and community based digital groups. Offline questionnaires were collected in person in Hefei, Anhui Province, and Nanjing, Jiangsu Province. In Hefei, participants were recruited primarily through community service centers and senior activity centers. In Nanjing, offline data were collected from university affiliated residential communities for faculty and staff families and from senior activity centers.

The questionnaire was developed on the basis of established scales and prior studies, with wording adapted to fit the context of older adults’ use of smart interactive products (Yin et al., 2024). Prior experience with smart interactive products was required because the central behavioral-engagement variables examined in this study, including usage frequency, functional diversity, and interactive depth, presuppose some actual experience with the technology. Therefore, the sampling criterion was designed to ensure that participants could provide meaningful evaluations of their engagement behaviors. This criterion also means that the present sample represents technology-experienced older adults rather than the entire older adult population, including complete non-users. Specifically, the measurement design drew on the Technology Acceptance Model for perceived usefulness, the self-efficacy tradition for technology self-efficacy, the Self-Determination Theory framework for psychological need satisfaction, the Satisfaction With Life Scale and the Positive and Negative Affect Schedule for subjective wellbeing, and recent digital literacy studies for the measurement of digital literacy. The full questionnaire is provided in Appendix 1.

The survey was administered through both online and offline channels to accommodate differences in digital access and response preferences among older adults (Khan et al., 2026). Online questionnaires were distributed through social platforms and community-based digital groups, whereas offline questionnaires were collected in person from older adults in community settings (Miller et al., 2024). A total of 2,686 questionnaires were initially collected, including 2,558 online questionnaires and 128 offline questionnaires. After data screening, 51 questionnaires were excluded because of incomplete or otherwise unusable responses. The final analytic sample therefore consisted of 2,635 valid questionnaires, including 2,507 online responses and 128 offline responses, yielding an effective response rate of 98.10%.

Participant characteristics were further summarized to provide a more transparent description of the analytic sample. As shown in Table 2, the 2,635 participants varied in age, gender, educational attainment, and duration of prior smart-product use. Among the participants, 1,602 (60.8%) were aged 60–69 years, 831 (31.5%) were aged 70–79 years, and 202 (7.7%) were aged 80 years or above. The sample included 1,287 men (48.8%) and 1,348 women (51.2%). Prior experience with smart interactive products also varied across participants: 292 respondents (11.1%) had used such products for less than 1 year, 825 (31.3%) had 1 to 3 years of experience, and 1,518 (57.6%) had more than 3 years of experience. Among the final valid responses, 2,507 (95.1%) were collected through online channels and 128 (4.9%) through offline administration. Overall, although all participants had prior experience with at least one type of smart interactive product, the sample showed substantial variation in demographic background and duration of technology-use experience.

**Table 2:** Demographic and technology-use characteristics of the participants.

Characteristic	Category	Number (n)	Percentage (%)
Age	60–69 years	1,602	60.8
	70–79 years	831	31.5
	over 80 years	202	7.7
Gender	Male	1,287	48.8
	Female	1,348	51.2
Education level	Primary school or below	421	16.0
	Junior middle school	763	29.0
	Senior high school	882	33.5
	College or above	569	21.6
Duration of prior smart-product use	less than 1 year	292	11.1
	1–3 year	825	31.3
	over 3 years	1,518	57.6
Survey mode	Online	2,507	95.1
	Offline	128	4.9
Total		2,635	100

Before the formal survey, participants were informed of the purpose of the study and assured that their responses would be used only for academic research. Participation was voluntary, and anonymity and confidentiality were guaranteed throughout the data collection process. For offline administration, trained researchers provided necessary instructions when participants had difficulty understanding individual items, but no substantive guidance was given that could influence their responses. This procedure helped ensure that the questionnaire could be completed accurately while minimizing response bias.

### 3.2 Measures

All constructs were measured using multi-item scales adapted from established instruments and prior studies, with minor wording revisions to fit the context of older adults’ use of smart interactive products. All items were rated on a five-point Likert scale ranging from 1 (“strongly disagree”) to 5 (“strongly agree”).

#### 3.2.1 Psychological perceptions

Psychological perceptions were conceptualized as a multidimensional construct comprising perceived usefulness, technology self-efficacy, and technology anxiety (Niu et al., 2026). Perceived usefulness assessed the extent to which older adults believed that smart interactive products could improve daily life, facilitate communication, or support health management. Technology self-efficacy captured respondents’ confidence in their ability to learn and use smart interactive products effectively. Technology anxiety reflected feelings of nervousness, discomfort, or concern when interacting with such technologies. These three dimensions jointly represented older adults’ overall psychological perceptions toward smart interactive products.

#### 3.2.2 Behavioral engagement

Behavioral engagement was modeled as a second-order construct composed of three dimensions: usage frequency, functional diversity, and interactive depth (Kim and Choudhury, 2021). Usage frequency measured how often respondents used smart interactive products in daily life. Functional diversity assessed the extent to which they used different functions of smart interactive products for multiple purposes (Roquet et al., 2024). Interactive depth captured the degree to which respondents moved beyond basic operation and engaged in more active exploration of product functions and features. This multidimensional operationalization was adopted to distinguish sustained and meaningful engagement from simple technology access or occasional use.

#### 3.2.3 Psychological need satisfaction

Psychological need satisfaction was measured based on Self-Determination Theory and included three dimensions: autonomy, competence, and relatedness. Autonomy referred to the extent to which using smart interactive products helped older adults feel more independent in everyday life (Sen et al., 2022). Competence captured feelings of capability and accomplishment derived from successfully using smart technologies. Relatedness assessed whether smart interactive products helped respondents maintain communication and social connection with others. Together, these dimensions reflected the extent to which technology engagement contributed to the fulfillment of basic psychological needs.

#### 3.2.4 Subjective wellbeing

Subjective wellbeing was conceptualized as a higher-order construct reflecting both cognitive and affective evaluations of life (Xin et al., 2025). It included life satisfaction, positive affect, and negative affect. Life satisfaction assessed respondents’ overall evaluation of their life conditions, whereas positive affect and negative affect captured the emotional experiences associated with daily life (Li et al., 2026). Following common practice, negative affect items were reverse-coded so that higher values consistently indicated higher levels of subjective wellbeing.

#### 3.2.5 Digital literacy

Digital literacy was measured as the extent to which respondents felt capable of understanding and using digital technologies in everyday life (Hepburn et al., 2025). The construct focused on practical familiarity, operational confidence, and perceived ability to learn new digital tools. In the proposed model, digital literacy was treated as a moderating variable influencing the relationship between psychological perceptions and behavioral engagement.

#### 3.2.6 Control variables

To reduce potential confounding effects, demographic characteristics including age, gender, education level, and prior experience with smart interactive products were included as control variables in the analysis.

### 3.3 Data analysis

All statistical analyses were conducted using IBM SPSS Statistics 29.0 and IBM SPSS Amos 29.0. SPSS was used for descriptive statistics, reliability analysis, correlation analysis and one-way analysis of variance. Amos was used for confirmatory factor analysis (CFA), structural equation modeling (SEM), and measurement invariance testings. The mediation analysis was conducted using 5,000 bootstrap resamples to estimate the indirect effects and their 95% confidence intervals.

The analysis proceeded in four stages. First, descriptive statistics were calculated to summarize the sample characteristics and the basic distribution of the study variables. Reliability was then assessed using Cronbach’s alpha and composite reliability (CR). Second, confirmatory factor analysis (CFA) was conducted to evaluate the measurement model. Convergent validity was assessed based on standardized factor loadings, average variance extracted (AVE), and CR, whereas discriminant validity was examined by comparing the square root of AVE for each construct with the correlations among constructs.

Third, the structural model was tested to examine the hypothesized relationships among the latent variables. Model fit was evaluated using commonly recommended indices, including the comparative fit index (CFI), Tucker–Lewis index (TLI), root mean square error of approximation (RMSEA), and standardized root mean square residual (SRMR). Fourth, the mediating effect proposed in the TEW model was examined using bootstrapping with 5,000 resamples to estimate indirect effects and their confidence intervals. The moderating effect of digital literacy was tested by estimating the interaction effect between psychological perceptions and digital literacy on behavioral engagement. Control variables, including age, gender, education level, and prior experience with smart interactive products, were included in the analysis to reduce potential confounding effects.

To further examine heterogeneity among older adults, additional subgroup analyses were conducted according to educational attainment and technology-use frequency. For educational attainment, participants were classified into lower-, secondary-, and higher-education groups. Differences in the major study variables were first examined using one-way analysis of variance, followed by multi-group structural equation modeling to assess whether the core structural relationships of the TEW model remained stable across educational groups. Measurement invariance was examined before structural-path comparisons. Participants were also classified into low-, moderate-, and high-frequency technology-use groups. Because usage frequency constitutes one dimension of behavioral engagement, comparisons across frequency groups focused on perceived usefulness, technology self-efficacy, technology anxiety, functional diversity, interactive depth, psychological need satisfaction, subjective wellbeing, and digital literacy. To avoid using technology-use frequency simultaneously as both a grouping criterion and a component of behavioral engagement, an additional robustness model was estimated in which behavioral engagement was represented by functional diversity and interactive depth only.

## 4 Results

### 4.1 Descriptive statistics and reliability

Descriptive statistics for the key constructs are presented in Table 3. The mean scores for all constructs were generally high, indicating that respondents viewed smart interactive products positively, felt confident in using them, and experienced meaningful psychological need satisfaction. Specifically, Perceived Usefulness and Psychological Need Satisfaction had the highest mean scores, reflecting participants’ beliefs in the utility of smart technologies and their ability to fulfill autonomy, competence, and relatedness needs. In contrast, Technology Anxiety had the lowest mean score, indicating that while some anxiety was reported, it did not dominate the overall responses. And the reliability analysis was conducted using Cronbach’s alpha to assess the internal consistency of the scales. All constructs exhibited high internal consistency, with Cronbach’s alpha values exceeding 0.80 for each scale. These values indicate that the measurement scales used in this study are reliable and consistent across respondents.

**Table 3:** Descriptive statistics and reliability for key constructs.

Construct	*M*	SD	Cronbach’s Alpha
Perceived usefulness (PU)	4.229	0.741	0.841
Technology self-efficacy (TSE)	4.109	0.800	0.850
Technology anxiety (TA)	2.469	1.019	0.870
Behavioral engagement (BE)	4.101	0.780	0.917
Psychological need satisfaction (PNS)	4.321	0.688	0.926
Subjective wellbeing (SWB)	4.259	0.720	0.923
Digital literacy (DL)	4.150	0.808	0.856

M, Mean; SD, Standard Deviation.

The correlations were consistent with the proposed model, Table 4 presents the correlation coefficients between the key variables. Perceived usefulness and technology self-efficacy were positively correlated with behavioral engagement, psychological need satisfaction, subjective wellbeing, and digital literacy. Technology anxiety was negatively correlated with the other key constructs. Behavioral engagement was positively associated with psychological need satisfaction (*r* = 0.598, *p* < 0.001) and subjective wellbeing (*r* = 0.501, *p* < 0.001). Psychological need satisfaction was also positively associated with subjective wellbeing (*r* = 0.641, *p* < 0.001).

**Table 4:** Correlations between constructs.

Construct	1	2	3	4	5	6	7
Perceived usefulness (PU)	1						
Technology Self-Efficacy (TSE)	0.530***	1					
Technology anxiety (TA)	−0.343***	−0.327***	1				
Behavioral engagement (BE)	0.388***	0.449***	−0.168***	1			
Psychological need satisfaction (PNS)	0.599***	0.619***	−0.282***	0.598***	1		
Subjective wellbeing (SWB)	0.538***	0.457***	−0.352***	0.501***	0.641***	1	
Digital literacy (DL)	0.480***	0.499***	−0.234***	0.418***	0.547***	0.465***	1

***p < 0.001.

### 4.2 Measurement model

To assess the validity of the measurement model, Confirmatory Factor Analysis (CFA) was conducted using Structural Equation Modeling (SEM). The CFA was performed to evaluate the fit of the measurement model and ensure that each construct had adequate factor loadings and discriminant validity. The measurement model showed an excellent fit to the data, with *χ*^2^ = 1161.708, df = 838, χ^2^/df = 1.386, CFI = 0.995, TLI = 0.995, and RMSEA = 0.012. These results indicate that the latent measurement structure was well supported by the observed data.

All standardized factor loadings were statistically significant (*p* < 0.001) and ranged from 0.724 to 0.804, indicating adequate item reliability. Table 5 presents the standardized loading ranges, composite reliability (CR), and average variance extracted (AVE) for each construct. Standardized factor loadings ranged from 0.723 to 0.805. CR values ranged from 0.841 to 0.926, and all AVE values exceeded the recommended threshold of 0.50, indicating adequate convergent validity.

**Table 5:** Reliability and convergent validity of the measurement model.

Construct	Standardized loading range	CR	AVE
Perceived usefulness (PU)	0.742–0.762	0.841	0.569
Technology self-efficacy (TSE)	0.745–0.779	0.850	0.586
Technology anxiety (reverse-coded)	0.777–0.805	0.870	0.625
Behavioral engagement (BE)	0.724–0.748	0.915	0.546
Psychological need satisfaction (PNS)	0.750–0.773	0.926	0.583
Subjective wellbeing (SWB)	0.733–0.751	0.917	0.551
Digital literacy (DL)	0.756–0.787	0.856	0.599

Discriminant validity was assessed by comparing the square root of the Average Variance Extracted (AVE) for each construct with the correlations between constructs. As shown in Table 6, the square root of the AVE for each construct was greater than its correlations with any other construct, providing evidence of adequate discriminant validity. Overall, the CFA results support the reliability and validity of the measurement model.

**Table 6:** Discriminant validity.

Construct	PU	TSE	TA(r)	BE	PNS	SWB	DL
PU	0.754						
TSE	0.530	0.766					
TA(r)	0.343	0.327	0.791				
BE	0.388	0.449	0.168	0.739			
PNS	0.599	0.619	0.282	0.598	0.763		
SWB	0.538	0.457	0.352	0.501	0.641	0.742	
DL	0.480	0.499	0.234	0.418	0.547	0.465	0.774

Diagonal elements are the square roots of AVE.

### 4.3 Structural model

After confirming the adequacy of the measurement model, the structural model was tested to examine the hypothesized relationships among constructs. As shown in Table 7, the structural model showed a good fit to the data, *χ*^2^ = 2230.041, df = 852, *χ*^2^/df = 2.618, CFI = 0.979, TLI = 0.977, and RMSEA = 0.025.

**Table 7:** Fit indices of the structural model.

Fit index	Value
*χ* ^2^	2230.041
df	852
*χ*^2^/df	2.618
CFI	0.979
TLI	0.977
RMSEA	0.025

The results showed that psychological perceptions had a significant positive effect on behavioral engagement (*β* = 0.651, *z* = 22.836, *p* < 0.001), supporting H1. Behavioral engagement also had a significant positive effect on psychological need satisfaction (*β* = 0.255, *z* = 29.338, *p* < 0.001), supporting H2. Psychological need satisfaction, in turn, had a significant positive effect on subjective wellbeing (*β* = 0.422, *z* = 19.750, *p* < 0.001), supporting H3. The strongest path was from psychological perceptions to behavioral engagement, followed by the path from psychological need satisfaction to subjective wellbeing. This pattern indicates that older adults’ evaluations of smart interactive products were closely associated with whether they engaged with these technologies, and that engagement was strongly related to the satisfaction of autonomy, competence, and relatedness needs.

As shown in Table 8, psychological perceptions were positively associated with behavioral engagement (*β* = 0.651, *p* < 0.001) and psychological need satisfaction (*β* = 0.441, *p* < 0.001). Behavioral engagement was positively associated with psychological need satisfaction (*β* = 0.255, *p* < 0.001) and subjective wellbeing (*β* = 0.115, *p* < 0.001), while psychological need satisfaction was positively associated with subjective wellbeing (*β* = 0.422, *p* < 0.001). The direct association between psychological perceptions and subjective wellbeing remained significant after the mediators were included (*β* = 0.290, *p* < 0.001). These results provide the structural coefficients required for the specific indirect effects examined in the subsequent mediation analysis.

**Table 8:** Structural path coefficients and hypothesis testing.

Path	Standardized coefficient (*β*)	*p*-value	Result
Psychological perceptions → behavioral engagement	0.651	<0.001	H1 supported
Psychological perceptions → psychological need satisfaction	0.441	< 0.001	Mediation component
Psychological perceptions → subjective wellbeing	0.290	< 0.001	Direct effect retained
Behavioral engagement → psychological need satisfaction	0.255	<0.001	H2 supported
Behavioral engagement → subjective wellbeing	0.115	< 0.001	Mediation component
Psychological need satisfaction → subjective wellbeing	0.422	< 0.001	H3 supported

### 4.4 Mediation analysis

To test the proposed serial mediation mechanism, the mediating role of behavioral engagement and psychological need satisfaction was tested using bootstrap estimation with 5,000 resamples. The total effect of psychological perceptions on subjective wellbeing was significant (*β* = 0.621). After the mediators were included, the direct effect remained significant [*β* = 0.290, 95% CI (0.251, 0.333)], indicating that the relationship was not fully explained by the indirect path.

The sequential indirect effect of psychological perceptions on subjective wellbeing through behavioral engagement and psychological need satisfaction was significant [*β* = 0.070, 95% CI (0.058, 0.084)]. In addition to this serial path, the indirect effect through behavioral engagement alone was significant [*β* = 0.075, 95% CI (0.055, 0.096)], and the indirect effect through psychological need satisfaction alone was also significant [*β* = 0.186, 95% CI (0.156, 0.214)]. The total indirect effect was 0.331 [95% CI (0.297, 0.364)]. Because the confidence intervals for all indirect effects did not include zero, H4 was supported. The Bootstrap results for the mediation analysis was shown in Table 9.

**Table 9:** Bootstrap results for the mediation analysis.

Effect	*β*	95% CI	Result
Total effect	0.621	—	Significant
Direct effect	0.290	[0.251, 0.333]	Significant
Indirect effect: PP → BE → SWB	0.075	[0.055, 0.096]	Significant
Indirect effect: PP → PNS → SWB	0.186	[0.156, 0.214]	Significant
Indirect effect: PP → BE → PNS → SWB	0.070	[0.058, 0.084]	Significant
Total indirect effect	0.331	[0.297, 0.364]	Significant

PP, psychological perceptions; BE, behavioral engagement; PNS, psychological need satisfaction; SWB, subjective wellbeing.

### 4.5 Moderation analysis

The moderating effect of digital literacy on the relationship between psychological perceptions and behavioral engagement was tested by including the interaction term between psychological perceptions and digital literacy. Psychological perceptions had a significant positive effect on behavioral engagement (*β* = 0.390, *t* = 17.380, *p* < 0.001), and digital literacy also had a significant positive effect on behavioral engagement (*β* = 0.358, *t* = 19.187, *p* < 0.001). More importantly, the interaction term between psychological perceptions and digital literacy was significant (*β* = 0.390, *t* = 17.907, *p* < 0.001), supporting H5.

As shown in Table 10, a simple slope analysis showed that the effect of psychological perceptions on behavioral engagement was weaker at lower levels of digital literacy (*β* = 0.075) and stronger at higher levels of digital literacy (*β* = 0.705). This pattern indicates that older adults with higher digital literacy were more likely to translate favorable psychological perceptions of smart interactive products into actual behavioral engagement.

**Table 10:** Moderation analysis results.

Predictor	Coefficient (*β*)	*t*-value	*p*-value
Psychological perceptions	0.390	17.380	<0.001
Digital literacy	0.358	19.187	<0.001
Psychological perceptions × digital literacy	0.390	17.907	<0.001

### 4.6 Subgroup and heterogeneity analyses

#### 4.6.1 Educational-background subgroup analysis

To examine whether the structural relationships proposed in the TEW model differed according to educational background, participants were classified into three groups: lower education (primary school or below and junior middle school, *n* = 1,184), secondary education (senior high school or vocational education, *n* = 882), and higher education (college education or above, *n* = 569). Before comparing structural paths, measurement invariance was examined across the three groups. The configural model showed satisfactory fit (CFI = 0.978, TLI = 0.976, RMSEA = 0.026), indicating that the same general measurement structure was supported across educational groups. The metric-invariance model also showed satisfactory fit (CFI = 0.977, TLI = 0.976, RMSEA = 0.026), with ΔCFI = 0.001, supporting the comparability of structural coefficients across groups.

As shown in Table 11, the core structural relationships of the TEW model remained significant across all three educational groups. Psychological perceptions positively predicted behavioral engagement, behavioral engagement positively predicted psychological need satisfaction, and psychological need satisfaction positively predicted subjective wellbeing in each group. Although the magnitude of the coefficients varied moderately across educational levels, none of the between-group differences in the three core structural paths reached statistical significance. These results indicate that educational attainment may be associated with differences in older adults’ technology-related characteristics, but the principal engagement–need satisfaction–wellbeing mechanism remained comparatively stable across educational groups.

**Table 11:** Multi-group SEM results across educational levels.

Structural path	Lower education *β*	Secondary education *β*	Higher education *β*	Between-group *p*
Psychological perceptions → Behavioral engagement	0.621***	0.649***	0.674***	0.084
Behavioral engagement → Psychological need satisfaction	0.661***	0.683***	0.697***	0.291
Psychological need satisfaction → Subjective wellbeing	0.407***	0.426***	0.441***	0.417

***p < 0.001.

#### 4.6.2 Technology-use-frequency subgroup analysis

To further examine heterogeneity associated with actual technology use, participants were classified into low-, moderate-, and high-frequency technology-use groups according to their usage-frequency scores. Because usage frequency is itself one component of behavioral engagement, the complete behavioral engagement score was not used as a comparison variable in this analysis. Instead, comparisons focused on perceived usefulness, technology self-efficacy, technology anxiety, functional diversity, interactive depth, psychological need satisfaction, subjective wellbeing, and digital literacy.

As shown in Table 12, systematic differences were observed across technology-use-frequency groups. Higher-frequency users reported greater perceived usefulness, stronger technology self-efficacy, greater functional diversity and interactive depth, and higher digital literacy, together with lower technology anxiety. Psychological need satisfaction and subjective wellbeing also increased across frequency groups, although the corresponding effect sizes were smaller than those observed for functional diversity, interactive depth, and digital literacy. These findings indicate that technology-use frequency is particularly associated with the breadth and depth of engagement and with older adults’ technology-related capability.

**Table 12:** Psychological and behavioral profiles across technology-use-frequency groups.

Variable	Lower frequency M (SD)	Moderate frequency M (SD)	High frequency M (SD)	*F*	*p*	η^2^
Perceived usefulness	3.98 (0.80)	4.22 (0.72)	4.43 (0.64)	54.62	<0.001	0.040
Technology self-efficacy	3.74 (0.88)	4.10 (0.77)	4.45 (0.65)	106.34	<0.001	0.075
Technology anxiety	2.91 (1.05)	2.47 (0.97)	2.08 (0.88)	101.28	<0.001	0.071
Functional diversity	3.63 (0.82)	4.09 (0.71)	4.49 (0.62)	171.55	<0.001	0.115
Interactive depth	3.54 (0.86)	4.02 (0.73)	4.46 (0.65)	182.47	<0.001	0.122
Psychological need satisfaction	4.12 (0.73)	4.31 (0.67)	4.49 (0.61)	35.76	<0.001	0.026
Subjective wellbeing	4.12 (0.76)	4.26 (0.70)	4.39 (0.65)	19.61	<0.001	0.015
Digital literacy	3.72 (0.88)	4.15 (0.75)	4.53 (0.62)	150.28	<0.001	0.102

#### 4.6.3 Robustness analysis excluding usage frequency

Because usage frequency was used as the grouping criterion and also constitutes one dimension of behavioral engagement in the primary TEW model, an additional robustness analysis was conducted after excluding usage frequency from the behavioral engagement construct. In this alternative specification, behavioral engagement was represented by functional diversity and interactive depth only. The principal structural relationships remained statistically significant and consistent with those of the primary model in both direction and magnitude. Psychological perceptions continued to positively predict behavioral engagement, behavioral engagement positively predicted psychological need satisfaction, and psychological need satisfaction positively predicted subjective wellbeing. These findings indicate that the core TEW mechanism was not driven solely by the inclusion of usage frequency in the behavioral engagement construct.

## 5 Discussion

### 5.1 Main findings and interpretation

This study examined how older adults’ psychological perceptions of smart interactive products are translated into behavioral engagement and, ultimately, subjective wellbeing. The results support the core logic of the TEW model. Psychological perceptions significantly predicted behavioral engagement, behavioral engagement positively predicted psychological need satisfaction, and psychological need satisfaction enhanced subjective wellbeing. These findings suggest that the value of smart interactive products does not lie in access alone, but in whether positive perceptions can be converted into active engagement that supports autonomy, competence, and relatedness.

The strongest path in the model was from psychological perceptions to behavioral engagement. This finding is consistent with recent research showing that older adults’ use of digital technologies depends strongly on perceived usefulness, confidence, and related psychological barriers. For example, DeLange Martinez et al. (2024, 2025) found that perceived usefulness and perceived ease of use were associated with smartphone and ICT use among low-income Asian American older adults, while a later JMIR Aging study further suggested that improving these perceptions may also reduce loneliness through greater ICT acceptance. The present study extends this line of work by showing that psychological perceptions are not only relevant to acceptance or intention, but remain central in shaping actual behavioral engagement with smart interactive products.

The positive effect of behavioral engagement on psychological need satisfaction also adds an important layer to the literature. Recent work has increasingly treated digital engagement as more than simple access or binary use. Du et al. (2026), for example, modeled digital engagement as a structured mechanism and showed that its effects operated through mediators such as digital health literacy and social support. Our findings are consistent with this mechanism-based perspective, but they further show that engagement is psychologically consequential because it is associated with the satisfaction of autonomy, competence, and relatedness. This helps clarify why some forms of technology use are more beneficial than others: it is not technology exposure itself, but the quality and depth of engagement, that appear to matter.

The findings also align with previous evidence linking technology-related attitudes to wellbeing in later life. Atta et al. (2024) reported that gerontechnology acceptance was positively associated with mental wellbeing and that this relationship was partly explained by acceptance of change. Compared with this broader acceptance–wellbeing association, the present study offers a more explicit explanatory chain by showing that psychological perceptions are linked to wellbeing partly through behavioral engagement and subsequent psychological need satisfaction.

The moderating role of digital literacy is likewise theoretically meaningful. Recent studies indicate that digital participation among older adults is shaped not only by attitudes toward technology, but also by opportunities to build digital capability. Community-based digital literacy interventions have been shown to improve older adults’ participation and access, while recent work on digital anxiety and empowerment suggests that psychological barriers and digital capability develop together rather than independently. Our results extend this literature by showing that digital literacy functions as an enabling condition: positive psychological perceptions are more likely to translate into behavioral engagement when older adults possess stronger digital capability.

As shown in Table 13, recent studies have examined selected links among technology perceptions, digital engagement, literacy, and wellbeing in older adults, but few have integrated these elements into a single explanatory mechanism. The TEW model contributes by connecting these previously fragmented relationships into one coherent pathway.

**Table 13:** Comparison of the present study with representative prior studies on older adults’ technology use and wellbeing.

Prior study	Main focus	Main finding	What the present study adds
Atta et al. (2024)	Gerontechnology acceptance and mental wellbeing	Gerontechnology acceptance was positively associated with mental wellbeing through a psychological adaptation process.	Moves beyond a broad acceptance–wellbeing association by specifying a sequential pathway from psychological perceptions to engagement, need satisfaction, and subjective wellbeing.
DeLange Martinez et al. (2024, 2025)	Technology perceptions and ICT acceptance/use	Perceived usefulness and perceived ease of use were associated with ICT use and acceptance among older adults.	Extends this line by showing that psychological perceptions predict multidimensional behavioral engagement rather than acceptance alone.
Miller et al. (2024)	Digital literacy training among low-income older adults	Community-based digital literacy training improved older adults’ participation and access.	Shows that digital capability is not only trainable, but also functions as a boundary condition strengthening the perception–engagement link.
Du et al. (2026)	Digital engagement and mediated outcomes	Digital engagement influenced later outcomes through mediators such as digital health literacy and social support.	Identifies psychological need satisfaction as a distinct mechanism linking engagement to subjective wellbeing.
Niu et al. (2026)	Digital anxiety, empowerment, and literacy	Digital anxiety and sense of achievement were associated with technology-related use and literacy development.	Integrates anxiety-related barriers into a broader model that simultaneously explains engagement and wellbeing outcomes.
Present study	TEW model in smart interactive products	Psychological perceptions predicted behavioral engagement; engagement predicted psychological need satisfaction; need satisfaction predicted subjective wellbeing; digital literacy moderated the first-stage path.	Integrates psychological, behavioral, and wellbeing processes into one explanatory model for older adults’ smart interactive product use.

The subgroup analyses further indicate that older adults are heterogeneous in their technology-related characteristics. Educational attainment and technology-use frequency were particularly associated with technology self-efficacy, technology anxiety, digital literacy, and the depth and diversity of engagement. However, the downstream relationships linking behavioral engagement to psychological need satisfaction and subjective wellbeing were comparatively stable across educational groups. This distinction is theoretically important: demographic and experiential factors appear to influence how readily older adults engage with smart interactive products, whereas the psychological benefits associated with meaningful engagement may operate through a relatively consistent mechanism once engagement occurs. These findings therefore refine the TEW model by identifying heterogeneity primarily at the entry and engagement stages rather than suggesting that a single uniform behavioral profile characterizes all older adults.

### 5.2 Theoretical and practical implications

This study makes several contributions. First, it links psychological perceptions, behavioral engagement, psychological need satisfaction, and subjective wellbeing within one model, offering a clearer explanation of how smart interactive product use becomes relevant to wellbeing in later life. Much of the existing literature has either focused on technology acceptance and intention or examined broad associations between technology use and wellbeing. By integrating these components into a single framework, this study clarifies the mechanism through which smart interactive product use becomes beneficial.

Second, the study advances the conceptualization of behavioral engagement. Rather than treating technology use as a binary adoption outcome or a single frequency indicator, this study models engagement as a richer form of involvement that includes frequency, diversity, and interaction depth. This distinction matters because recent work has already suggested that digital engagement varies substantially in quality and meaning across older adults and that its effects operate through intermediate mechanisms rather than through use status alone. The present findings show that this richer form of engagement is closely tied to psychological need fulfillment.

Third, the study extends the relevance of Self-Determination Theory in the context of smart interactive products for older adults. The findings indicate that the contribution of technology to wellbeing is not merely functional. Products appear more beneficial when engagement with them enhances autonomy, competence, and relatedness. This point is also practically important. Design strategies should not focus only on usability or task completion, but also on whether products help older adults feel capable, in control, and socially connected.

The results further suggest that interventions aimed at increasing smart interactive product use among older adults should target psychological perceptions and digital capability at the same time. Improving perceived usefulness is important, but it is equally important to strengthen technology self-efficacy, reduce anxiety, and provide sufficient digital literacy support. Recent research shows that older adults’ technology acceptance is strongly shaped by perceived usefulness and related appraisal factors, while digital literacy interventions can improve participation and reduce exclusion. In practical terms, this means that age-friendly product strategies should combine inclusive design with accessible onboarding, clear feedback, and ongoing training or community support.

### 5.3 Limitations and future research

This study has several limitations. First, the cross-sectional design limits causal inference. Longitudinal or experimental research is needed to examine how changes in perception and engagement influence wellbeing over time. Second, the study relied on self-reported data. Future work could combine self-report measures with usage logs or task-based behavioral indicators. Third, the present sample was restricted to older adults who had prior experience with at least one type of smart interactive product. Because the TEW model examines behavioral engagement in terms of usage frequency, functional diversity, and interactive depth, all of which require some degree of prior technology use. Nevertheless, this sampling strategy limits the generalizability of the findings. The present results should therefore be interpreted primarily as evidence concerning technology-experienced older adults and should not be directly generalized to complete non-users or highly digitally marginalized populations. Older adults without prior technology experience may face qualitatively different barriers, including lack of access, limited exposure, physical or cognitive constraints, insufficient social support, or very low digital capability, before the engagement processes specified in the TEW model can occur. Future research should therefore test the model among non-users and digitally disadvantaged older adults and examine whether additional pre-engagement factors are required to explain the transition from non-use to initial adoption. Such work may further extend the TEW framework by linking pre-adoption barriers with the post-exposure engagement and wellbeing processes examined in the present study.

## 6 Conclusion

This study proposed and tested the Technology Engagement and Wellbeing (TEW) model to explain how older adults’ psychological perceptions of smart interactive products are associated with subjective wellbeing. The findings show that psychological perceptions significantly predict behavioral engagement, behavioral engagement promotes psychological need satisfaction, and psychological need satisfaction contributes to subjective wellbeing. Digital literacy further strengthens the relationship between psychological perceptions and behavioral engagement.

These results suggest that the wellbeing value of smart interactive products lies not in access alone, but in whether older adults can translate positive perceptions into meaningful engagement that supports autonomy, competence, and relatedness. By integrating psychological perceptions, behavioral engagement, psychological need satisfaction, and subjective wellbeing into one framework, this study provides a clearer explanation of how smart interactive product use becomes relevant to wellbeing in later life. The present findings primarily support the TEW model as an explanatory framework for older adults with prior technology-use experience; further validation is required before the model can be generalized to complete non-users or populations experiencing substantial digital exclusion. In conclusion, the TEW model offers both a useful framework for future research and a practical basis for the design of age-friendly smart technologies that better support older adults’ everyday lives and wellbeing.

## Data Availability

The original contributions presented in the study are included in the article/Supplementary material, further inquiries can be directed to the corresponding author.
