# Supplementary material for: Psychological perceptions to behavioral engagement: testing a mediated structural model of smart interactive product use and subjective wellbeing in older adults
Source: Front Psychol. 2026 Oct 28;17:1861208. doi: 10.3389/fpsyg.2026.1861208 (PMC13751454; doi:10.3389/fpsyg.2026.1861208)
Supplement: Supplementary file 1 [file Supplementary_file_1.docx]

Appendix 1. Survey Instruments

Part I. English version

Please answer all statements based on your actual experience of using smart interactive products. Unless otherwise indicated, all items are rated on a 5-point Likert scale.

Section A. Screening questions

| Code | Item | Response |
| --- | --- | --- |
| A1. | Are you 60 years old or above? | Yes / No |
| A2. | Have you used at least one smart interactive product (such as a smartphone, smart speaker, wearable device, or smart home device)? | Yes / No |

Section B. Psychological perceptions

| Code | Item | Response |
| --- | --- | --- |
| PU1 | Using smart interactive products improves my daily life. | 1 2 3 4 5 |
| PU2 | Smart interactive products are useful for managing everyday activities. | 1 2 3 4 5 |
| PU3 | Smart interactive products help me communicate more conveniently with others. | 1 2 3 4 5 |
| PU4 | Using smart interactive products makes some daily tasks easier to complete. | 1 2 3 4 5 |
| TSE1 | I feel confident in my ability to use smart interactive products. | 1 2 3 4 5 |
| TSE2 | I can learn how to use new smart product functions if I try. | 1 2 3 4 5 |
| TSE3 | I believe I can solve simple problems when using smart interactive products. | 1 2 3 4 5 |
| TSE4 | I am able to use smart interactive products without much difficulty. | 1 2 3 4 5 |
| TA1 | I feel nervous when using smart interactive products. | 1 2 3 4 5 |
| TA2 | I worry that I may make mistakes when using smart technologies. | 1 2 3 4 5 |
| TA3 | Using smart interactive products sometimes makes me feel uncomfortable. | 1 2 3 4 5 |
| TA4 | I feel anxious when I have to learn unfamiliar smart technologies. | 1 2 3 4 5 |

Section C. Behavioral engagement

| Code | Item | Response |
| --- | --- | --- |
| BEF1 | I frequently use smart interactive products in my daily life. | 1 2 3 4 5 |
| BEF2 | I use smart interactive products on a regular basis. | 1 2 3 4 5 |
| BEF3 | Smart interactive products are part of my everyday routine. | 1 2 3 4 5 |
| BED1 | I use different functions of smart interactive products for different purposes. | 1 2 3 4 5 |
| BED2 | I use smart interactive products for more than one type of daily activity. | 1 2 3 4 5 |
| BED3 | I make use of various features provided by smart interactive products. | 1 2 3 4 5 |
| BEI1 | I often explore new functions when using smart interactive products. | 1 2 3 4 5 |
| BEI2 | I try to learn more advanced ways of using smart interactive products. | 1 2 3 4 5 |
| BEI3 | My use of smart interactive products goes beyond only basic functions. | 1 2 3 4 5 |

Section D. Psychological need satisfaction

| Code | Item | Response |
| --- | --- | --- |
| AUT1 | Using smart interactive products makes me feel more independent in daily life. | 1 2 3 4 5 |
| AUT2 | Smart interactive products help me handle some daily tasks by myself. | 1 2 3 4 5 |
| AUT3 | I feel that smart interactive products give me more control over my daily activities. | 1 2 3 4 5 |
| COM1 | I feel capable when I successfully use smart interactive products. | 1 2 3 4 5 |
| COM2 | Learning to use smart interactive products gives me a sense of accomplishment. | 1 2 3 4 5 |
| COM3 | Using smart technologies makes me feel that I can keep up with modern life. | 1 2 3 4 5 |
| REL1 | Smart interactive products help me stay connected with family or friends. | 1 2 3 4 5 |
| REL2 | Using smart interactive products helps me feel less socially isolated. | 1 2 3 4 5 |
| REL3 | Smart interactive products make it easier for me to maintain contact with others. | 1 2 3 4 5 |

Section E. Subjective well-being

| Code | Item | Response |
| --- | --- | --- |
| SWL1 | In most ways, my life is close to ideal. | 1 2 3 4 5 |
| SWL2 | I am satisfied with my life overall. | 1 2 3 4 5 |
| SWL3 | The conditions of my life are good. | 1 2 3 4 5 |
| PA1 | In general, I often feel positive in daily life. | 1 2 3 4 5 |
| PA2 | I often feel cheerful or happy in my daily life. | 1 2 3 4 5 |
| PA3 | I often feel calm and content in everyday life. | 1 2 3 4 5 |
| NA1 | In general, I often feel upset in daily life. | 1 2 3 4 5 |
| NA2 | I often feel worried or stressed in my daily life. | 1 2 3 4 5 |
| NA3 | I often feel lonely or down in everyday life. | 1 2 3 4 5 |

Section F. Digital literacy

| Code | Item | Response |
| --- | --- | --- |
| DL1 | I feel comfortable using digital technologies in my daily life. | 1 2 3 4 5 |
| DL2 | I am able to learn how to use new digital tools. | 1 2 3 4 5 |
| DL3 | I can usually understand the basic functions of digital devices. | 1 2 3 4 5 |
| DL4 | I feel capable of dealing with common digital tasks on my own. | 1 2 3 4 5 |

Section G. Demographic information

| Code | Item | Response |
| --- | --- | --- |
| G1 | Gender | Male / Female / Prefer not to say |
| G2 | Age | 60-64 / 65-69 / 70-74 / 75-79 / 80 or above |
| G3 | Education level | Primary school or below / Junior high / Senior high or vocational / College diploma / Bachelor’s or above |
| G4 | Monthly income | Below 3,000 RMB / 3,000-4,999 / 5,000-6,999 / 7,000-9,999 / 10,000 or above |
| G5 | Smart interactive products used | Smartphone / Wearable device / Smart speaker or voice assistant / Smart home device / Online health app or device / Other |
| G6 | Duration of use | Less than 6 months / 6-12 months / 1-3 years / 3-5 years / More than 5 years |

Part II. 中文版问卷

请根据您使用智能交互产品的真实体验作答。除特别说明外，以下题项均采用 5 点李克特量表作答。

A部分 判断题

| Code | Item | Response |
| --- | --- | --- |
| A1 | 您的年龄是否为 60 岁及以上？ | 是 / 否 |
| A2 | 您是否使用过至少一种智能交互产品（如智能手机、智能音箱、可穿戴设备、智能家居设备等）？ | 是 / 否 |

B部分 心理感知

| Code | Item | Response |
| --- | --- | --- |
| PU1 | 使用智能交互产品能够改善我的日常生活。 | 1 2 3 4 5 |
| PU2 | 智能交互产品有助于我处理日常事务。 | 1 2 3 4 5 |
| PU3 | 智能交互产品能让我与他人沟通得更方便。 | 1 2 3 4 5 |
| PU4 | 使用智能交互产品能让一些日常任务更容易完成。 | 1 2 3 4 5 |
| TSE1 | 我对自己使用智能交互产品的能力有信心。 | 1 2 3 4 5 |
| TSE2 | 只要我愿意尝试，我能够学会使用新的智能产品功能。 | 1 2 3 4 5 |
| TSE3 | 我相信自己能够解决使用智能交互产品时遇到的一些简单问题。 | 1 2 3 4 5 |
| TSE4 | 我能够较为顺利地使用智能交互产品。 | 1 2 3 4 5 |
| TA1 | 使用智能交互产品时，我会感到紧张。 | 1 2 3 4 5 |
| TA2 | 使用智能技术时，我担心自己会出错。 | 1 2 3 4 5 |
| TA3 | 使用智能交互产品有时会让我感到不舒服。 | 1 2 3 4 5 |
| TA4 | 当我需要学习陌生的智能技术时，我会感到焦虑。 | 1 2 3 4 5 |

C部分 行为投入

| Code | Item | Response |
| --- | --- | --- |
| BEF1 | 在日常生活中，我经常使用智能交互产品。 | 1 2 3 4 5 |
| BEF2 | 我会较为规律地使用智能交互产品。 | 1 2 3 4 5 |
| BEF3 | 智能交互产品已经成为我日常生活的一部分。 | 1 2 3 4 5 |
| BED1 | 我会使用智能交互产品的不同功能来满足不同需求。 | 1 2 3 4 5 |
| BED2 | 我会将智能交互产品用于多种不同的日常活动。 | 1 2 3 4 5 |
| BED3 | 我会使用智能交互产品提供的多种功能。 | 1 2 3 4 5 |
| BEI1 | 使用智能交互产品时，我经常会主动探索新功能。 | 1 2 3 4 5 |
| BEI2 | 我会尝试学习更高级的智能交互产品使用方式。 | 1 2 3 4 5 |
| BEI3 | 我对智能交互产品的使用不仅限于基础功能。 | 1 2 3 4 5 |

D部分 心理需求满足

| Code | Item | Response |
| --- | --- | --- |
| AUT1 | 使用智能交互产品让我在日常生活中感到更独立。 | 1 2 3 4 5 |
| AUT2 | 智能交互产品帮助我自己完成一些日常事务。 | 1 2 3 4 5 |
| AUT3 | 我觉得智能交互产品让我对自己的日常活动有更多掌控感。 | 1 2 3 4 5 |
| COM1 | 当我成功使用智能交互产品时，我会感到自己很有能力。 | 1 2 3 4 5 |
| COM2 | 学会使用智能交互产品会给我带来成就感。 | 1 2 3 4 5 |
| COM3 | 使用智能技术让我觉得自己能够跟上现代生活。 | 1 2 3 4 5 |
| REL1 | 智能交互产品帮助我与家人或朋友保持联系。 | 1 2 3 4 5 |
| REL2 | 使用智能交互产品让我感觉不那么孤立。 | 1 2 3 4 5 |
| REL3 | 智能交互产品让维持与他人的联系变得更容易。 | 1 2 3 4 5 |

E部分 主观幸福感

| Code | Item | Response |
| --- | --- | --- |
| SWL1 | 在大多数方面，我的生活接近理想状态。 | 1 2 3 4 5 |
| SWL2 | 总体而言，我对自己的生活感到满意。 | 1 2 3 4 5 |
| SWL3 | 我的生活条件是良好的。 | 1 2 3 4 5 |
| PA1 | 总体而言，我在日常生活中经常感到积极。 | 1 2 3 4 5 |
| PA2 | 在日常生活中，我经常感到愉快或开心。 | 1 2 3 4 5 |
| PA3 | 在日常生活中，我经常感到平静和满足。 | 1 2 3 4 5 |
| NA1 | 总体而言，我在日常生活中经常感到烦躁或难受。 | 1 2 3 4 5 |
| NA2 | 在日常生活中，我经常感到担忧或压力。 | 1 2 3 4 5 |
| NA3 | 在日常生活中，我经常感到孤独或情绪低落。 | 1 2 3 4 5 |

F部分 数字素养

| Code | Item | Response |
| --- | --- | --- |
| DL1 | 在日常生活中使用数字技术时，我感到比较自如。 | 1 2 3 4 5 |
| DL2 | 我能够学会使用新的数字工具。 | 1 2 3 4 5 |
| DL3 | 我通常能够理解数字设备的基本功能。 | 1 2 3 4 5 |
| DL4 | 我觉得自己能够独立处理常见的数字操作任务。 | 1 2 3 4 5 |

G部分 人口统计信息

| Code | Item | Response |
| --- | --- | --- |
| G1 | 性别 | 男 / 女 / 不愿透露 |
| G2 | 年龄 | 60-64 岁 / 65-69 岁 / 70-74 岁 / 75-79 岁 / 80 岁及以上 |
| G3 | 受教育程度 | 小学及以下 / 初中 / 高中或中专 / 大专 / 本科及以上 |
| G4 | 月收入 | 3000 元以下 / 3000-4999 元 / 5000-6999 元 / 7000-9999 元 / 10000 元及以上 |
| G5 | 您使用过哪些智能交互产品？ | 智能手机 / 智能手表或可穿戴设备 / 智能音箱或语音助手 / 智能家居设备 / 在线健康管理应用或设备 / 其他 |
| G6 | 您使用智能交互产品的大约时长 | 6 个月以内 / 6-12 个月 / 1-3 年 / 3-5 年 / 5 年以上 |
